# Treatment of Malarial Hæmaturia

**Published:** 1891-04

**Authors:** B. W. Mason

**Affiliations:** Sheridan, Ark.


### TREATMENT OF MALARIAL HÆMATURIA.

By B. W. MASON, M. D., SHERIDAN, ARK.

In copy of JOURNAL for February I notice an article on the treatment of "Malarial Hæmaturia." The author of this article recommends the use of tincture iron mur. and Fowler's solution. Since I have been in Arkansas I have treated five cases of this disease; two of these cases were very bad, one died, the other three cases were seen early and all recovered. My treatment, was calomel, elixir, vitriol and ergot and buchu. Of these five cases only one was salivated, (very slightly); case recovered. While glancing over the JOURNAL that contained the above mentioned article, a gentleman came for me in great haste to see his child. I found the patient, a girl nine years old, who had been having chills for more than a year. Skin very yellow, pulse 135, temperature 102, bowels had been moved by a dose of salts, the bladder acting every hour or two, passing dark blood. The child complained of pain in head, back and bones of lower extremities. She was vomiting almost incessantly and the dyspnœa was so great that she had to be raised to a sitting posture and fanned frequently; this was to me an alarming symptom. I at once put her upon one grain doses of calomel every two hours until the bowels were acted upon ; for the nausea I gave Horsford's acid phosphate, which acted nicely. I gave tincture iron three drops, Fowler's solution one drop every two hours, and spirits turpentine four drops every two hours. This treatment was commenced Tuesday at 11 o'clock A. M. At 8 o'clock P. M. the urine began to look more natural, the skin to clear up, the nausea to subside. The last trace of blood in the urine disappeared at 9 o'clock A. M. Wednesday. Not a trace was to be discovered in discharge from the bladder two hours later. The patient began to improve and I discharged the case Saturday so far recovered as to sit up a little. I left a tonic for her and she made a quick and nice recovery. I think the iron and arsenic treatment will do.

